# The International Society of Urological Pathology Education web—a web-based system for training and testing of pathologists

**DOI:** 10.1007/s00428-019-02540-w

**Published:** 2019-02-21

**Authors:** Lars Egevad, Brett Delahunt, Hemamali Samaratunga, Katia RM Leite, Gennady Efremov, Bungo Furusato, Ming Han, Laura Jufe, Toyonori Tsuzuki, Zhe Wang, Jonas Hörnblad, Mark Clements

**Affiliations:** 1 Department of Oncology and pathology, Karolinska Institutet, Karolinska University Hospital, 171 76 Stockholm, Sweden; 2 Department of Pathology and Molecular Medicine, Wellington School of Medicine and Health sciences, University of Otago, Wellington, New Zealand; 3 Aquesta Uropathology and University of Queensland, Brisbane, QLD Australia; 4 Department of Urology, Laboratory of Medical Research, University of Sao Paulo Medical School, São Paulo, Brazil; 5 Department of Pathology, N. Lopatkin Scientific Research Institute of Urology and Interventional Radiology, Branch of the National Medical Radiological Research Centre of the Ministry of the Russian Federation, Moscow, Russia; 6 Department of Pathology, Nagasaki University Graduate School of Biomedical Sciences, Sakamoto, Nagasaki, Japan; 7 Department of Pathology, Xijing Hospital and School of Basic Medicine, Fourth Military Medical University, Xi’an, People’s Republic of China; 8 Division Anatomia Patologica, Hospital JM Ramos Mejia, Buenos Aires, Argentina; 9 Department of Surgical Pathology, School of Medicine, Aichi Medical University, 1-1 Yazakokarimata, Nagakute, Japan; 10 Department of Medical Epidemiology and Biostatistics, Karolinska Institutet, Stockholm, Sweden

**Keywords:** Pathology, Standardization, Database, Grading, Diagnosis, Prostate, Kidney, Bladder

## Abstract

Pathology training resources remain scarce in many parts of the world. With rapid economic development comes the need to educate new pathologists to meet the medical care demands. Our aim was to set up a cost-effective system for training and testing the diagnostic skills of pathologists. Pathologists in nine countries in Asia and South America were invited by the International Society of Urological Pathology (ISUP) to participate in a prostate pathology education course combining image-based tests with lectures and on-line tutorials. The tests and tutorials are available free of charge at the ISUP education website www.edu.isupweb.org. A total of 603 pathologists registered on the website. Of these, 224 completed pre- and post-lecture assessments (tests 1 and 2). Replies were classified as correct/acceptable, when a lesion was accurately classified into clinically relevant categories (benign, cancer, high-grade prostatic intraepithelial neoplasia, intraductal carcinoma of the prostate). The rate of correct/acceptable replies increased from 60.7 to 72.3% in Tests 1 and 2, respectively. In Test 1, pathologists from upper middle, lower middle, and low resource countries gave a correct/acceptable diagnosis in 65.8%, 61.0%, and 47.4%, respectively. Their results improved in Test 2 to 76.4%, 72.5%, and 62.8%, respectively. The greatest improvement in diagnostic ability was achieved in pathologists from the low resource group of countries. The use of web-based testing and training, combined with lectures, is an efficient method for improving diagnostic skills of pathologists in low to middle resource countries.

## Introduction

Pathologists have traditionally been trained through a combination of course-based teaching and personal tutoring in daily practice. Both methods are labor intensive for teaching staff, and often pathology courses are didactic and lacking an interactive component. The practice of tertiary level medicine is evolving internationally, and with the current, rapid economic development of lower resource countries, pathology services are now expected to be provided in regions where high-level training may not be available.

We describe a novel mechanism for low-cost self-tutoring in urological pathology, developed under the auspices of the International Society of Urological Pathology (ISUP). The project was launched to improve the standard of prostate biopsy pathology interpretation and reporting, with an emphasis on the accurate diagnosis of prostate cancer and its mimics. The purpose of this study was to evaluate the efficiency of this form of teaching of pathologists from low or middle resource countries.

## Material and methods

**Fig. 1:**
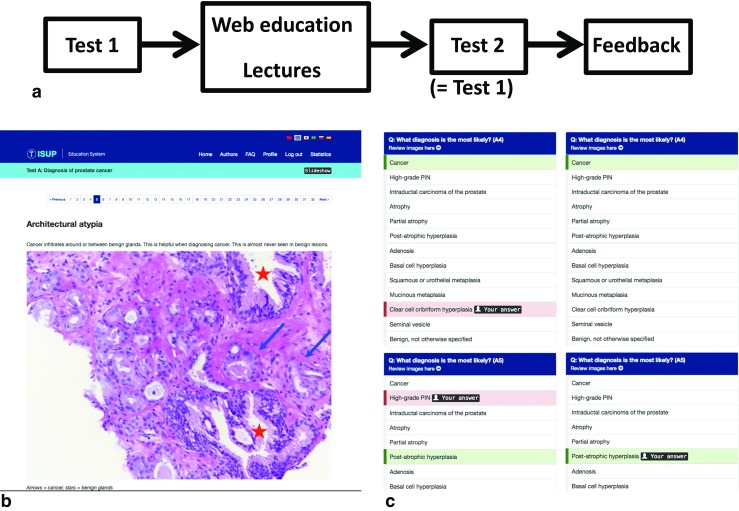
**a** Study design of the ISUP education project. Participants were asked to do two identical image-based tests with an intervening slideshow and lectures. The correct replies were not disclosed until after the second test was completed. **b** Example of slideshow figure with brief text and explanatory arrows and asterisks. **c** Feedback page released after the completion of both tests. Results of the two tests appear side by side with correct, acceptable, and incorrect responses color-coded in green, yellow, and red, respectively. Links to test images are provided

In the autumn of 2016, pathologists from nine countries in South America and Southeast Asia were invited to participate in an education program focusing on prostate pathology. To assess the impact of economic development on prostate pathology training, we used the World Bank Classification of the Economic Status of Countries (2012) (Table 1) in the analysis [1]. Five of the countries included in the study were listed as upper middle, two as lower middle, and two as low resource countries. For privacy reasons, the names of the countries will not be disclosed. The training program was offered free of charge at www.edu.isupweb.org. It can also be accessed through the ISUP website (www.isupweb.org). Participants were required to register with a username and an email address to obtain access to the web-based program and to the assessments. The email addresses were used to provide reminders for participants to complete the tests and to circulate feedback to participants at a group level. At registration, the participants were asked to specify their professional status as pathologist (specialist), pathologist (resident/trainee), clinician, basic scientist, or other. The pathologists (specialist and resident/trainees) were asked to provide details of their professional activity in pathology. These data consisted of number of years in practice including their residency, broken down into groups of 1–5, 6–10, 11–15, 16–20, or more than 20 years. The country in which they practiced was also registered. The study design is illustrated in Fig. 1. As an evaluation of the educational efficiency of the program, two identical tests were undertaken before and after the period of training.

**Table 1:** Participating countries by World Bank income class and number of participants completing both tests. The income classes were upper middle (UM), lower middle (LM), and lower (L) resource countries

Country	Income class	Participants (*n*)
A	UM	6
B	UM	7
C	UM	32
D	UM	56
E	UM	20
F	LM	13
G	LM	42
H	L	41
I	L	7
Total		224

Between the assessments, the participants participated in lectures and a web-based slideshow tutorial. None of the test microphotographs were presented in the on-line slideshow or at the lectures. The first assessment was closed the day before the lectures were presented, while the tutorial and the second assessment were made available immediately upon the completion of the lectures. The lectures were delivered by a single presenter (L.E.) and included comprehensive presentations on the diagnostic features of prostate cancer and its mimics, and also on contemporary issues relating to grading, while the assessments evaluated the ability to recognize prostate cancer and its mimics. The lectures were given in 12 cities during October and November 2016.

**Fig. 2:**
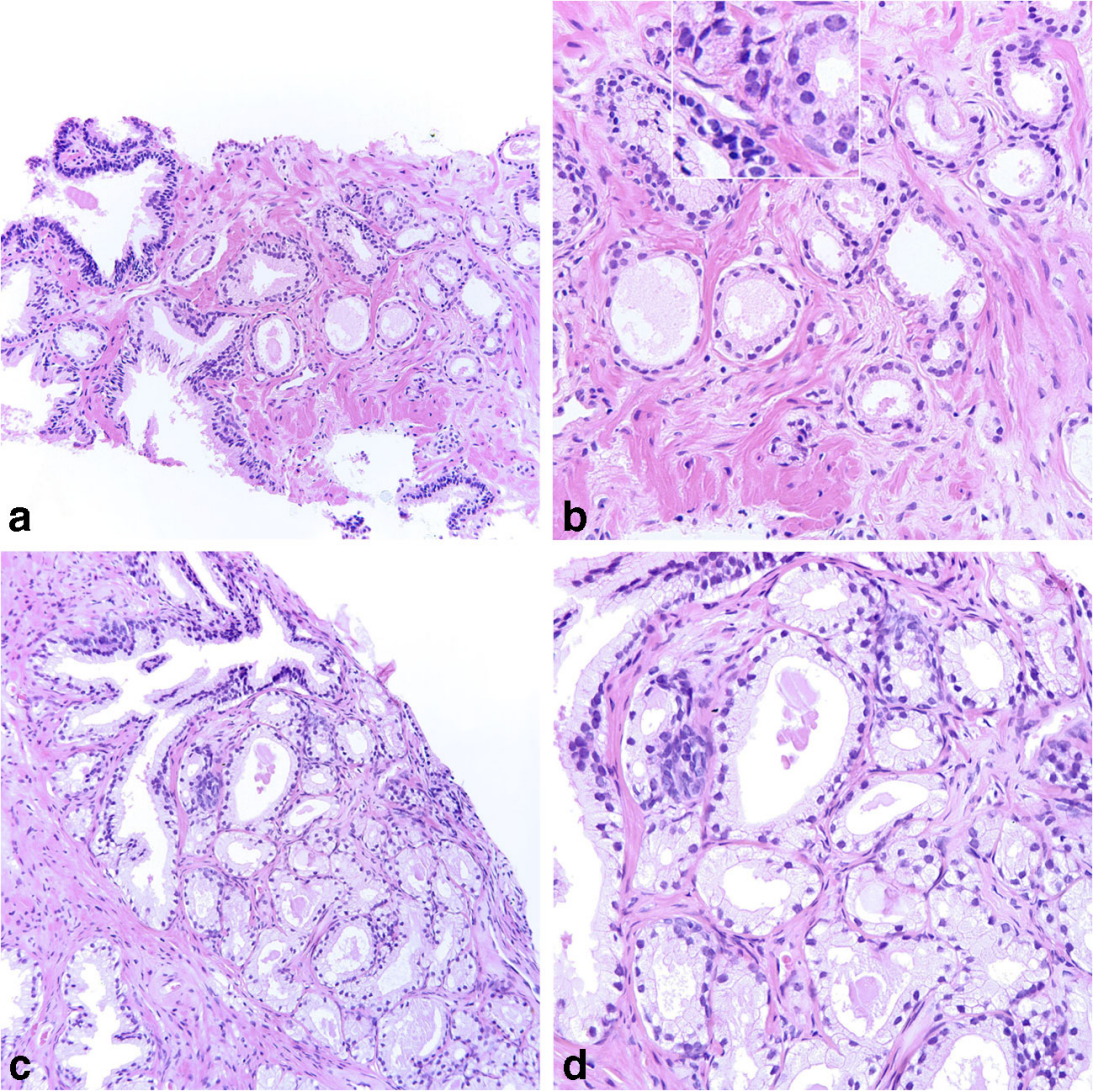
Examples of images from the test. **a**, **b** Prostate cancer shown at × 20 and × 40 lens magnification. **b** The magnifying tool shows a higher magnification of a square area at the top of the field, facilitating the identification of nuclear atypia. The rate of correct or acceptable replies was low, probably because of the mild atypia, but increased from 27.7% in Test 1 to 57.1% in Test 2. **c**, **d** Adenosis shown at × 20 and × 40 lens magnification. The rate of correct or acceptable replies increased from 79.5% in Test 1 to 89.3% in Test 2

The assessments consisted of 16 cases, including 5 cancers and 11 cancer mimics (adenosis, atrophy, basal cell hyperplasia, clear cell hyperplasia, partial atrophy, post-atrophic hyperplasia, and seminal vesicle). Each of the cases were presented in the form of 3–7 (mean 5) microphotographs in jpg format illustrating the lesion at different magnifications (Fig. 2a–d). The jpg images had a resolution of 72 dpi and a size of 20 × 20 cm or 25 × 25 cm. When moving the pointer over the images, a magnifying lens appeared, showing a higher magnification of that particular area (Fig. 2b). The participants cast their votes as to the diagnosis by selecting one of the following options from a multiple-choice menu: cancer, high-grade PIN, intraductal carcinoma of the prostate, atrophy, partial atrophy, post-atrophic hyperplasia, adenosis, basal cell hyperplasia, squamous or urothelial metaplasia, mucinous metaplasia, clear cell cribriform hyperplasia, and seminal vesicle and benign, not otherwise specified. All cancer diagnoses were verified by immunohistochemistry for p63 and alpha-methylacyl-CoA racemase, clone p504s (Ventana), although no immunohistochemical stained sections were available on the website. The participants were asked to state the diagnosis they thought was the most likely based on the hematoxylin and eosin-stained illustrations only. Thus, with atypical glands suspicious for cancer, but with features insufficient for a conclusive diagnosis, the correct reply would be cancer.

The diagnosis of the various benign lesions of the prostate may cause difficulties in routine practice. For example, it can be somewhat arbitrary where the line is drawn between atrophy, partial atrophy, and postatrophic hyperplasia as diagnostic entities. This has, however, little or no clinical implications, and for this reason, the replies were classified as correct (C), acceptable (A), and false or incorrect (F), where correct/acceptable refers to a correct classification by the clinically relevant categories benign, cancer, high-grade PIN, and intraductal carcinoma of the prostate. Thus, any benign reply was considered acceptable even if the correct response was another benign diagnosis. In the analyses and presentation of the results, correct or acceptable replies were grouped and compared against incorrect replies.

After completion of both assessments, immediate feedback was given on-line (Fig. 1c) and the results of the two tests were shown side by side. Thus, for this study, no feedback was given after Test 1. The results of Test 1 were locked as soon as the respondent started the slideshow, and the results of Test 2 were locked when feedback had been given. After the completion of the current study, this mechanism has been modified to allow respondents to choose if they want to see the correct replies after Test 1 or if they also want to do Test 2. If they decide to see the feedback document after Test 1, then access to Test 2 is denied. Correct, acceptable, or incorrect replies were color-coded in green, yellow, and red, respectively. There was an option to review the images again, but once the assessments were completed, the replies could not be modified.

A mobile telephone-friendly version of the website was also established and the website was further developed by adding translations of slideshows and assessments in Chinese, Japanese, Portuguese, Russian, and Spanish. The link to the website was distributed to countries in South America and Asia where lectures had not been given in the first phase of this study. For this component of the study, responses were only received from upper middle resource countries. A total of 84 pathologists completed both assessments. Results were compared against those of upper middle resource countries in the lecture phase of the study.

### Statistical analyses

A cancer lesion was defined as a true positive if the cancer was identified. A benign lesion was defined as a true negative if the lesion was identified in either (a) the correct category or (b) the correct or an acceptable category. Test *accuracy* was defined as the proportion of all lesions that were classified as true positives or true negatives. Sensitivity was defined as the proportion of cancer lesions that were classified as true positive. Specificity was defined as the proportion of benign lesions that were classified as true negatives.

Test accuracy was defined as the average proportion of replies that were (a) correct or (b) either correct or acceptable. These measures were interpreted as weighted sums of sensitivity and specificity. For a single test, we estimated the proportion of tests that were accurate with binomial confidence intervals, and calculated the relative accuracy and confidence intervals using methods for paired data [2]. The proportion of tests that were accurate at Test 1 or Test 2 were modeled using log-binomial regression. Relative accuracy was defined as the test accuracy at Test 2 divided by the test accuracy at Test 1. Relative accuracy was estimated using generalized estimating equations with a binomial distribution and a log link [3]. We tested for heterogeneity in relative accuracy by covariates using an interaction model with main effects for time of test (i.e., Test 1 vs. 2) and covariate and an interaction between time and the covariate. Measures of sensitivity, specificity, relative sensitivity, and relative specificity used similar methods.

## Results

In total, 603 individuals registered on the web site. Division of attendees according to professional groups showed 554 participants to be pathologists or residents/trainees in pathology. The other professional categories included clinicians and basic scientists that were excluded from further analysis, and only pathologists or residents/trainees are included in the data presented below. Participants who had more than one missing value in each of the assessments were also excluded from the analysis.

A single missing value was considered an incorrect reply for analytical purposes. The number of participants who completed Tests 1 and 2 with no more than one missing value in each test was 387 and 224, respectively (Table 1). In light of this, only the 224 complete tests were further evaluated as this permitted paired analyses and avoided any bias caused by including responses submitted by less motivated individuals who only completed Test 1.

Among the 224 respondents who practiced pathology, 118 were specialists, 102 were residents/trainees, and 4 did not specify their level of education in pathology. Of the specialists, 21 had practiced pathology for 1–5 years, 35 for 6–10 years, or 17 for 11–15 years, 23 for 16–20 years, and 16 for > 20 years, while 6 did not report the duration of their careers in pathology.

Overall, the rate of correct/acceptable replies improved from 60.7% (2174/3584) to 72.2% (2586/3584) from Test 1 to Test 2 with a relative accuracy of 1.19 (95% confidence interval [CI] 1.14–1.25) (Table 2). The rate of totally correct replies improved from 31.8% (1141/3584) to 48.0% (1721/3584) from Test 1 to Test 2, with a relative accuracy of 1.51 (95% CI 1.37–1.66).

**Table 2:** Results in Tests 1 and 2 for all participants and for specialists and residents/trainees. Percent correct/acceptable replies in %, numbers, and relative accuracy

	Test 1		Test 2		
Category	% (95% CI)	*n* (95% CI)	% (95% CI)	*n* (95% CI)	Relative accuracy (95% CI)
Specialists	63.3 (61.2–65.6)	10.1 (9.8–10.5)	75.8 (73.9–77.8)	12.1 (11.8–12.4)	1.20 (1.13–1.27)
Residents/trainees	57.3 (54.9–59.7)	9.2 (8.8–9.6)	67.5 (65.3–69.8)	10.8 (10.4–11.2)	1.18 (1.10–1.26)
Total	60.7 (59.1–62.3)	9.7 (9.5–10.0)	72.2 (70.7–73.6)	11.5 (11.3–11.8)	1.19 (1.14–1.25)

Both specialists and residents/trainees improved their results from Test 1 to Test 2 with relative accuracies of 1.20 (CI 1.13–1.27) and 1.18 (CI 1.10–1.26), respectively. Specialists had 63.3% (1196/1888) and 75.8% (1432/1888) correct or acceptable replies in Tests 1 and 2, respectively, vs. 57.3% (935/1632) and 67.5% (1102/1632) for residents/trainees (Table 2). We found no evidence that the relative accuracy between tests for specialists was different to that for residents or trainees (*p* = 0.74). In both Test 1 and Test 2, specialists tended to be more accurate in their diagnoses than residents or trainees, with relative accuracies of 1.11 (95% CI 1.05–1.17) and 1.12 (95% CI 1.08–1.17), respectively.

The correct or acceptable rate varied among the countries evaluated in the study ranging from 45.7 to 77.1% in Test 1 and from 58.0 to 84.4% in Test 2 (Table 3). Six of nine countries showed a significant improvement in the second test with the greatest improvement being from 45.7 to 62.5% correct answers. We found moderate evidence for heterogeneity in the relative accuracy between countries (*p* = 0.02).

**Table 3:** Results by country. Percent correct/acceptable replies in % (95% CI) and number (95% CI). The income classes were upper middle (UM), lower middle (LM), and lower (L) resource countries

	Test 1		Test 2		
Country	% (95% CI)	*n* (95% CI)	% (95% CI)	*n* (95% CI)	Relative accuracy (95% CI)
A	76.8 (69.4–85.0)	12.3 (11.1–13.6)	83.0 (76.4–90.3)	13.3 (12.2–14.4)	1.08 (0.86–1.36)
B	57.1 (48.7–67.1)	9.1 (7.8–10.7)	58.0 (49.6–67.9)	9.3 (7.9–10.9)	1.02 (0.73–1.41)
C	76.8 (73.2–80.5)	12.3 (11.7–12.9)	84.4 (81.3–87.6)	13.5 (13.0–14.0)	1.10 (1.02–1.19)
D	59.5 (56.4–62.8)	9.5 (9.0–10.0)	71.5 (68.6–74.6)	11.4 (11.0–11.9)	1.20 (1.10–1.31)
E	45.7 (42.1–49.7)	7.3 (6.7–8.0)	62.5 (58.9–66.3)	10.0 (9.4–10.6)	1.37 (1.22–1.54)
F	58.4 (53.3–64.1)	9.4 (8.5–10.3)	74.4 (69.7–79.3)	11.9 (11.2–12.7)	1.27 (1.11–1.46)
G	62.6 (59.1–66.4)	10.0 (9.5–10.6)	73.1 (69.8–76.5)	11.7 (11.2–12.2)	1.17 (1.07–1.27)
H	77.1 (69.1–86.0)	12.3 (11.1–13.8)	76.0 (68.0–85.1)	12.2 (10.9–13.6)	0.99 (0.86–1.14)
I	55.8 (49.4–62.9)	8.9 (7.9–10.1)	68.8 (62.7–75.3)	11.0 (10.0–12.1)	1.23 (1.03–1.47)

The study showed that the higher the economic class of the country, the better was the performance of its pathologists (Table 4). In Test 1, pathologists from upper middle, lower middle, and low resource countries gave a correct/acceptable diagnosis in 65.8%, 61.0%, and 47.4% of cases, respectively. Although not part of the study design, tests identical to Test 1 have been used in high resource countries in North America, Europe, and South East Asia with a total of 132 respondents by September 2017 and correct or acceptable diagnoses in 75.6 to 83.7%. The results in Test 2 were respectively 76.4%, 72.5%, and 62.8%. Although the improvement in the rate of correct diagnoses was greater in low resource countries, a trend towards better results with higher economic class remained in the second test. The performance gap between the upper middle to low resource countries, i.e., the difference in their rates of correct/acceptable diagnoses, decreased from 18.4 to 14.6%. We found little evidence for heterogeneity in the relative accuracy between countries by income class for the classroom-based teaching (*p* = 0.19). Restricting the analysis to upper-middle-income countries, we found that the web-based delivery was associated with a similar test accuracy for correct/acceptable replies at Test 1 compared with classroom-based teaching (relative accuracy = 0.98, 95% CI 0.93–1.03). The relative accuracy from Test 1 to Test 2 for web-based delivery was 1.11 (95% CI 1.03–1.19), which was lower, but not significantly different (*p* = 0.32), to that for classroom-based teaching (1.19; see Table 2).

**Table 4:** Results by income class. Percent correct/acceptable replies in % (mean, range) and number (mean, range). The income classes were upper middle (UM), lower middle (LM), and lower (L) resource countries

	Test 1		Test 2		
Countries by income class	% (95% CI)	*n* (95% CI)	% (95% CI)	*n* (95% CI)	Relative accuracy (95% CI)
UM (non-web)	65.8 (63.7–67.9)	10.5 (10.2–10.9)	76.3 (74.4–78.2)	12.2 (11.9–12.5)	1.16 (1.10–1.23)
LM (non-web)	61.0 (57.9–64.3)	9.8 (9.3–10.3)	72.0 (69.1–75.1)	11.5 (11.1–12.0)	1.18 (1.09–1.27)
L (non-web)	47.4 (44.0–51.1)	7.6 (7.0–8.2)	61.8 (58.5–65.4)	9.9 (9.4–10.5)	1.30 (1.16–1.46)
UM (web)	64.1 (61.6–66.8)	10.3 (9.9–10.7)	71.0 (68.6–73.5)	11.4 (11.0–11.8)	1.11 (1.03–1.19)

For all test questions, except for a case of partial atrophy, there was an improvement of both completely correct and correct/acceptable answers between the tests (Table 5). There was a wide range of correct or acceptable reporting in both Test 1 (17.0–91.5%) and Test 2 (20.5–95.5%). Among benign diagnoses, the correct identification of partial atrophy was particularly challenging, but a majority of respondents at least identified the lesions as benign. The diagnoses which presented little difficulty were basal cell hyperplasia, seminal vesicle, and clear cell cribriform hyperplasia. It was also noted that the diagnosis of some cancers proved to be difficult, even in the follow-up second assessment, due to the presence of minimal atypia, cutting artifacts, or low number of atypical glands (Fig. 2).

**Table 5:** Results by case in Tests 1 and 2. Percent exactly correct and correct or acceptable replies in percent and number

		Test 1		Test 2	
Case	Diagnosis	% correct (95% CI)	% correct or acceptable (95% CI)	% correct (95% CI)	% correct or acceptable (95% CI)
1	Adenosis	26.3 (20.7–32.6)	79.5 (73.6–84.6)	50.0 (43.3–56.7)	89.3 (84.5–93.0)
2	Cancer	36.6 (30.3–43.3)	36.6 (30.3–43.3)	52.2 (45.5–58.9)	52.2 (45.5–58.9)
3	Partial atrophy	27.2 (21.5–33.6)	78.6 (72.6–83.8)	24.6 (19.1–30.7)	77.2 (71.2–82.6)
4	Cancer	27.7 (21.9–34.0)	27.7 (21.9–34.0)	57.1 (50.4–63.7)	57.1 (50.4–63.7)
5	Postatrophic hyperplasia	27.7 (21.9–34.0)	67.4 (60.8–73.5)	45.1 (38.5–51.9)	75.0 (68.8–80.5)
6	Clear cell cribriform hyperplasia	55.4 (48.6–62.0)	86.2 (80.9–90.4)	61.6 (54.9–68.0)	91.5 (87.1–94.8)
7	Seminal vesicle	61.2 (54.4–67.6)	69.6 (63.2–75.6)	92.9 (88.7–95.9)	95.5 (91.9–97.8)
8	Basal cell hyperplasia	62.9 (56.3–69.3)	72.3 (66.0–78.1)	79.9 (74.1–85.0)	84.8 (79.4–89.3)
9	Partial atrophy	12.5 (8.5–17.6)	70.5 (64.1–76.4)	26.3 (20.7–32.6)	78.6 (72.6–83.8)
10	Cancer	50.9 (44.1–57.6)	50.9 (44.1–57.6)	79.0 (73.1–84.2)	79.0 (73.1–84.2)
11	Partial atrophy	11.2 (7.4–16.0)	71.0 (64.6–76.8)	22.8 (17.4–28.8)	71.9 (65.5–77.7)
12	Postatrophic hyperplasia	25.9 (20.3–32.1)	76.8 (70.7–82.1)	45.1 (38.5–51.9)	83.5 (78.0–88.1)
13	Cancer	21.0 (15.8–26.9)	21.0 (15.8–26.9)	38.4 (32.0–45.1)	38.4 (32.0–45.1)
14	Partial atrophy	7.6 (4.5–11.9)	91.5 (87.1–94.8)	11.6 (7.7–16.5)	89.3 (84.5–93.0)
15	Cancer	17.0 (12.3–22.5)	17.0 (12.3–22.5)	20.5 (15.4–26.4)	20.5 (15.4–26.4)
16	Adenosis	38.4 (32.0–45.1)	54.0 (47.3–60.7)	61.2 (54.4–67.6)	70.5 (64.1–76.4)

For completely correct diagnoses, the relative sensitivity between Test 1 and Test 2 was 1.64 and the relative specificity was 1.46 (Table 6). However, when grouping the replies in clinically relevant categories (benign, cancer, high-grade PIN, and intraductal carcinoma of the prostate), the relative specificity was lower at 1.11, with 74.3% in Test 1 and 82.5% in Test 2.

**Table 6:** Results by diagnosis in Tests 1 and 2. Percent exactly correct and correct or acceptable replies with relative sensitivity or specificity (with 95% CI). The results for cancer are sensitivities and relative sensitivity. The results for benign are specificities and relative specificity between Test 1 and Test 2. The results for cancer for “Correct or acceptable” are as for the results for “Correct”

	Correct			Correct or acceptable		
	Test 1	Test 2	Relative sensitivity/specificity	Test 1	Test 2	Relative sensitivity/specificity
Cancer	30.6 (28.0–33.4)	49.5 (46.6–52.5)	1.62 (1.44–1.81)			
Benign	32.4 (30.6–34.3)	47.4 (45.4–49.4)	1.46 (1.36–1.58)	74.3 (72.6–76.1)	82.5 (81.0–84.0)	1.11 (1.07–1.15)

## Discussion

The provision of quality pathology diagnostic services is currently one of the great international challenges in healthcare. It is an erroneous assumption that cancer is mainly a disease of the industrialized world, as cancer incidence has increased significantly in most countries since 1990 [4]. It has been estimated that among new cancer cases, the proportion diagnosed in less developed countries will increase from 56% of all global cancer in 2008 to over 60% in 2030 [5]. Concern has been raised that this increasing incidence will be more challenging for developing countries because of their lack of healthcare infrastructure and resources [4]. In some African countries, there is only one pathologist per 1–5 million inhabitants [6, 7], while in some parts of Asia, there is also a severe shortage of pathologists. For example, in 2013, only seven pathologists were in practice in Cambodia with a population of 14 million, approximately 250 pathologists were in Vietnam with 90 million inhabitants, and there were approximately 400 pathologists in Indonesia with 223 million inhabitants (personal communication). Although the number of pathologists has increased in some of these countries since 2013, a serious deficiency will, no doubt, persist for many years ahead. This is also reflected in the lower participation of pathologists from low and lower middle resource countries compared to upper resource countries. In parallel with this, the resources required to educate new pathologists will exceed the teaching capacity of senior academic and professional staff. In addition to this, lack of resources limits participation in international congresses and courses further reducing educational opportunities. Clearly, experienced experts with good teaching skills lack the resources to visit every region in need, and this problem is compounded by security issues associated with some countries and the existence of a language barrier, which itself is an impediment to educational activities.

Large-scale projects to promote the clinical diagnosis of oral and cervical cancer at low cost have been launched in low resource countries, and their efficiency has been proven [8, 9]. These projects were aimed to provide simple tests, with marginally lower sensitivity, to the many, rather than exact but expensive tests for the few. For standardization of pathology reporting, we have recently established a web-based educational tool for tumor grading and classification. This program, known as Imagebase, consists of expert-vetted cases collected into a digital image repository [10, 11]. Web-based collections of images are commonly used in pathology education especially in undergraduate teaching. While repositories of images do promote uniformity of reporting, there remains a need for the production of basic educational material to develop diagnostic skills. To our knowledge, this is the first attempt to establish a mechanism to facilitate low-cost systematic training of pathologists in low to middle resource countries.

We have presented a novel, web-based mechanism for training and assessment in histopathology, which is accessible as a free program for pathologists world-wide. The profession of the pathologist is based on visual pattern recognition, and for this reason, all the assessments in the program are entirely image-based. We also recognized that pathologists in low resource countries may not have access to high-speed internet connections and so we chose to utilize jpg images rather than digitized slides. Another advantage of the use of microphotographs is the speed with which the pathologist can read the images. The slide tutorials were deliberately kept simple with very brief written statements to accompany the images, which were marked with arrows and asterisks. To make the testing and training set readily available to pathologists from countries where English is not well understood, all tests were translated into other major languages.

For the evaluation of the web training, assessments relating to the diagnosis of prostate cancer were established and we believe this to be an important and timely development. The incidence of prostate cancer is now increasing in areas of the world where the disease was only occasionally diagnosed. The increase in awareness of the clinical importance of prostate cancer will naturally lead to an increase in the number of prostate biopsies being undertaken. As this develops, it is imperative that local pathologists broaden their skills to keep pace with the increasing complexity of prostate cancer diagnosis and treatment.

This study has demonstrated a correlation between diagnostic performance and the World Bank classification of the economy of the countries where the pathologists practice. In the first assessment participants in low resource countries managed to accurately identify 47.4% of cases as benign or malignant, which is a result less than that expected from random distribution. However, the greatest improvement in the second assessment was seen in these countries, which may, in part, be explained by the very limited exposure of the participants to prostate biopsies prior to undertaking the course.

It must be emphasized that the results of this program do not translate into general performance in clinical practice. The benign differential diagnoses in the test series were on the average more challenging than the average benign biopsy encountered in general diagnostic pathology. Similarly, the cancers were also more diagnostically challenging than the typical carcinomas seen on needle biopsies of a variety of organs. In addition, a web-based test may be more difficult to interpret. In routine practice, pathologists will utilize real glass slides giving a greater resolution than on-screen images. They also have the option of examining multiple levels of a lesion as well as immunohistochemically stained sections. Our assessments should only be interpreted as a measure of the efficiency of training program, not a predictor of performance in general clinical practice.

In conclusion, we have demonstrated that the use of on-line teaching and testing in histopathology has the potential to improve the performance of pathologists. It is clear that alternative teaching methods, utilizing novel communication platforms, will play an increasing role in the future education of medicine. It is imperative that professional organizations such as ISUP take a lead role in the development of these tools in order to promote a high teaching standard and ultimately facilitate improvement in the practice of pathology internationally.
